# Sensitization to workplace respiratory allergens among bakery workers in Douala, Cameroon: a cross-sectional study

**DOI:** 10.1186/s13223-015-0080-2

**Published:** 2015-04-10

**Authors:** Bertrand Hugo Mbatchou Ngahane, Francis Nde, Eliane Ngomo, Emmanuel Afane Ze

**Affiliations:** Internal Medicine, Douala General Hospital, PO Box 4856, Douala, Cameroon; Ecole de Santé Publique, Université Libre de Bruxelles, Bruxelles, Belgium; Université des Montagnes, Bangangte, Cameroon; Faculty of Medicine and Biomedical Sciences, University of Yaounde 1, Yaounde, Cameroon; Faculty of Medicine and Pharmaceutical Sciences, University of Douala, Douala, Cameroon; Douala Research Network, Douala, Cameroon

**Keywords:** Asthma, Wheat allergen, α-amylase, Allergen sensitization, Bakeries

## Abstract

**Background:**

Sensitization to flour or fungal alpha-amylase is a prerequisite for the development of respiratory allergy in bakers. The knowledge of occupational allergen sensitization among bakery workers will facilitate the implementation of preventive measures for respiratory allergies in bakeries. The objective of this study was to determine the prevalence and factors associated with sensitization to wheat flour and α-amylase in bakers in Douala.

**Methods:**

A cross-sectional study was conducted in 42 of the 151 bakeries that are present in the city of Douala. Demographics, clinical data, as well as results of skin prick tests to wheat flour, α-amylase and common aeroallergens were collected from all participants. A logistic regression model of the SPSS.20 software was used to identify factors associated with sensitization to wheat flour and α-amylase.

**Results:**

Of the 229 participants included in the study, 222 (96.9%) were male. The mean age was 36.3 ± 8.9 years. The prevalence of sensitization to flour and α-amylase were 16.6% and 8.3% respectively. After multivariate analysis, factors associated with sensitization to flour were work seniority and sensitization to storage mites while an age of 30 years and above was the only factor associated with sensitization to α-amylase.

**Conclusion:**

Bakers in Douala are at risk of sensitization to occupational allergens. The environmental hygiene in bakeries, health surveillance and the use of personal protective equipment could reduce the risk of respiratory allergies among bakers.

## Background

Respiratory allergy in bakers is one of the main occupational respiratory diseases [1,2]. Asthma and allergic rhinitis are clinical expressions of respiratory allergy. In bakers, the prevalence of asthma varies from 5 to 21% and that of allergic rhinitis from 3 to 27% [3]. Although the mechanisms of baker’s asthma are not completely understood, IgE-mediated response (Gell and Coombs type I) is the major pathogenic mechanism responsible for the induction of work-related symptoms in wheat-exposed workers [4]. This IgE response is demonstrated by positive skin tests and the detection of serum-specific IgE antibodies to wheat flour allergens in individuals exposed to flour.

The main cause of respiratory allergy in bakers is flour which can be made of wheat, rye or barley [5,6]. In Cameroon, wheat flour is the most commonly used. It contains a complex mixture of multiple allergens [7]. Other allergens implicated in respiratory allergy in bakers are enzymes used as additives. The α-amylase of fungal origin is the most known common enzyme, the others being fungal xylanase and cellulase [8,9].

Many authors have studied the prevalence of sensitization to inhalant allergens present in professional bakeries. In Europe and Canada, the prevalence of sensitization to wheat flour is between 4 and 47% while that of sensitization to α-amylase varies from 4.6 to 24% [10]. In Africa, studies of sensitization of occupational airborne allergens in bakers are scarce. In Morocco, Alaoui et al. found 14.5% of bakers were sensitized to wheat flour [11]. In South Africa, Baatjes et al. noted a prevalence of sensitization to wheat flour in bakers at 16% and 3% for the α-amylase [12]. Several epidemiological studies have investigated the determinants of sensitization to bakery-related occupational aeroallergens. The results of these studies showed that atopy is the main determinant [10]. Other associated factors are the exposure to these airborne allergens and work seniority [13-15]. This study aimed to determine the prevalence and determinants of sensitization to flour and alpha-amylase in bakers in a resource limited setting.

## Methods

### Design

This was a cross-sectional study conducted from May 1st to July 31st 2013 in 42 randomly selected bakeries in Douala.

### Study setting

The study took place in the city of Douala, economic capital of Cameroon, a country located in Central Africa. Douala is a seaside city which hosts many industries. The climate is hot and humid with temperatures ranging from 21°C to 31°C and humidity ranging from 81% to 89% during the year.

### Study participants

We randomly selected 42 factory bakeries among the 151 found in the city of Douala. All the bakeries were factory bakeries producing bread and cakes. In each bakery, all the bakers aged 15 years or above and regularly in contact with flour were invited to participate in the study. Bakery employees regularly in contact with flour were included. Participants currently on antihistamines and those not consenting were excluded.

### Data collection and variables

Each worker who agreed to participate in the study was interviewed. Sociodemographic characteristics, past medical history, respiratory symptoms, family history of atopy, smoking status and duration of daily exposure to flour were collected. Smokers were participants smoking at least weekly. Ex-smokers were those who had stopped smoking for at least six months. All others were considered non-smokers. Rhinitis was defined by the presence of at least one of the following symptoms: sneezing, itchy nose, nasal obstruction and rhinorrhea. Rhinoconjunctivitis was defined by the association of symptoms of allergic, ocular itching or eye tearing. For the investigation of asthma, we used items of in Douala the questionnaire of the International study of asthma and allergies in childhood [16]. Work-related asthma symptoms and work-related rhinitis were respectively asthma symptoms and rhinitis triggered or aggravated at the workplace and improved when away from work.

Skin prick tests (SPT) to 13 aeroallergens were performed using workplace and common aeroallergens from ALK Laboratories (Argonne in Varennes, France). Workplace aeroallergens tested were wheat flour, fungal α-amylase and 4 species of storage mites *(Lepidoglyphus destructor, Glycyphagus domesticus, Acarus siro, Tyroglyphus putrescentia)*. The common aeroallergens tested were dust mites *(Dermatophagoides farinae, Dermatophagoides pteronyssinus)*, molds *(Alternaria alternata, Aspergillus fumigatus),* cat dander, dog dander and German cockroach.

The positive control was histamine and glycerosaline solution was used as negative control. Wheal diameters were measured 15 to 20 minutes after testing. The value recorded was the mean of the 2 perpendicular measures. The test was considered positive when the mean wheal diameter was greater (or equal to) 3 mm [17]. We defined atopy in this study as a positive skin test to at least one of the common aeroallergens.

### Statistical analysis

Data entry and data analysis were performed using SPSS software (Statistical Package for Social Scientists version 20.0, SPSS, Inc., Chicago, IL). Categorical variables were summarized as frequencies and percentages, while continuous data were presented as the median with the interquartile range. For the identification of factors associated with sensitization to workplace aeroallergens, we had 2 outcomes of interest: the sensitization to wheat flour and the sensitization to fungal α-amylase. The potential factors associated with the outcomes were: age, gender, smoking, family atopy, daily duration of exposure to flour, work seniority, atopy and sensitization to storage mites (positive SPT to at least one of the 4 storage mites).

A univariate analysis was performed using chi-square test. Multivariate logistic regression analysis was used to estimate the independent effects of different factors on the outcome variable. All the variables for which the p-value was less than (or equal to) 0.2 after the univariate analysis were introduced in the model for multivariate analysis. The adjusted odds ratios and their 95% confidence intervals (95% CI) were determined. Statistical tests were considered significant for a P value of less than 0.05.

### Ethical clearance

The study protocol was given ethical approval by the National Ethics Committee and verbal consent was obtained from each participant before the recruitment.

## Results

During the study period, 273 bakery employees had been invited to participate in the study and 229 of them had agreed to be included, giving a response rate of 83.8%.

Table 1 shows the baseline characteristics of the study population. The median age of participants was 35 years with an interquartile (IQR) range of 30 and 42 years. Male sex was the most represented gender with 222 participants (96.9%). The median duration of work seniority was 10 years (IQR: 5 – 15) while the prevalence of smoking was 24.5%.

**Table 1:** **Baseline characteristics of participants**

**Variables**	**Number**	**Percentage**
	**N = 229**	
Gender		
Male	222	96.9%
Female	7	3.1%
Age (years)		
Median (IQR)	35 (30 – 42)	
20–29	56	24.5%
30–39	99	43.2%
40–49	49	21.4%
50–59	25	10.9%
Work seniority (years)		
Median (IQR)	10 (5 – 15)	
Daily exposure (hours)		
Median (IQR)	9 (8 – 10)	
≤8	104	45.4%
>8	125	54.6%
Smoking		
Yes	55	24%
Ex smoker	7	3.1%
No smoking	167	71.9%
Symptoms of asthma		
Cough during the night	59	25.8%
Ever wheezing	9	3.9%
Ever wheezing during exercise	8	3.5%
Work related wheezing	6	2.6%
Rhinitis		
Yes	56	24.5%
No	173	75.5%
Work related rhinitis	35	15.3%
Rhinoconjuntivitis	15	6.6%

Table 2 shows the proportions of subjects with a positive SPT to different aeroallergens. They were positive in 51.5% of cases. The prevalence of sensitization to wheat flour and α-amylase were respectively 16.6% (95% CI 11.8 - 21.4) and 8.3% (95% CI 4.8 - 12.2). Sensitization to storage mites was found in 28 participants (12.2%: 95% CI 8.3 - 16.6) while the prevalence of atopy was 39.7% (95% CI 33.6 - 45.9).

**Table 2:** **Prevalence of atopy and specific sensitization to aeroallergens**

**Sensitization**	**Number**	**Percentage**
	**N = 229**	
Atopy (common aeroallergens)	91	39.7%
Wheat flour	38	16.6%
α-amylase	19	8.3%
Wheat flour or α-amylase	51	22.3%
At least one storage mite	28	12.2%
*Lepidoglyphus destructor*	8	3.5%
*Glycyphagus domesticus*	6	2.6%
*Acarus siro*	10	4.4%
*Tyroglyphus putrescentia*	11	4.8%
At least one dust mite	29	12.7%
*Dermatophagoides farinae*	18	7.9%
*Dermatophagoides pteronyssinus*	14	6.1%
At least one mould	23	10%
*Alternaria alternata*	14	6.1%
*Aspergillus fumigatus*	12	5.2%
Cockroach	34	14.8%
Dog dander	23	10%
Cat dander	19	8.3%

The results of the univariate analysis (Table 3) revealed that work seniority, atopy and sensitization to storage mites were associated with wheat flour sensitization. Meanwhile, age greater than 30 years, atopy and sensitization to storage mites were associated with α-amylase sensitization.

**Table 3:** **Univariate analysis of factors associated with workplace allergens**

**Variables**	**Sensitization to wheat flour**			**Sensitization to α-amylase**		
	**Yes**	**No**	**P-value**	**Yes**	**No**	**P-value**
Age						
>30 years	31 (19.6%)	127 (80.4%)	0.06	18 (11.4%)	140 (88.6%)	0.01
≤30 years	7 (9.9%)	64 (90.1%)		1 (1.4%)	70 (98.6%)	
Gender						
Male	38 (17.1%)	184 (82.9%)	0.6	18 (8.1%)	204 (91.9%)	0.45
Female	0 (0%)	7 (100%)		1 (14.3%)	6 (85.7%)	
Smoking						
Yes	11 (20%)	44 (80%)	0.43	4 (7.3%)	51 (92.7%)	0.75
No	27 (15.5%)	147 (84.5%)		15 (8.6%)	159 (91.4%)	
Family atopy						
Yes	7 (30.4%)	16 (69.6%)	0.07	2 (8.7%)	21 (91.3%)	0.9
No	31 (15%)	175 (85%)		17 (8.30)	189 (91.7%)	
Daily exposure to flour						
≤8 hours	17 (16.3%)	87 (83.7%)	0.9	12 (11.5%)	92 (88.5%)	0.10
>8 hours	21 (16.8%)	104 (83.2%)		7 (5.6%)	118 (94.4%)	
Work seniority						
≤5 years	2 (4.3%)	45 (95.7%)		2 (4.3%)	45 (95.7%)	
6 – 10 years	16 (19.3%)	67 (80.7%)	0.03	6 (7.2%)	77 (92.8%)	0.5
>10 years	20 (20.2%)	79 (79.8%)	0.02	11 (11.1%)	88 (88.9%)	0.19
Storage mite sensitization						
Yes	11 (39.3%)	17 (60.7%)	0.001	6 (21.4%)	22 (78.6%)	0.01
No	27 (13.4%)	174 (86.6%)		13 (6.5%)	188 (93.5%)	
Atopy						
Yes	21 (23.1%)	70 (76.9%)	0.03	12 (13.2%)	79 (86.8%)	0.02
No	17 (12.3%)	121 (87.7%)		7 (5,1%)	131 (94.9%)	
Sensitization to α-amylase						
Yes	6 (31.6%)	13 (68.4%)	0.09	-		-
No	32 (15.2%)	178 (84.8%)				
Wheat flour sensitization						
Yes	-	-	-	6 (15.8%)	32 (84.2%)	0.09
No				13 (6.8%)	178 (93.2%)	

After multivariate analysis (Table 4), the factors that were independently associated with sensitization to wheat flour were work seniority, atopy and sensitization to storage mites. Age greater than 30 years was the only factor independently associated with α-amylase sensitization.

**Table 4:** **Multivariate analysis of factors associated with workplace aeroallergens**

**Variables**	**Sensitization to wheat flour**		**Sensitization to α-amylase**	
	**aOR (95% CI)**	**P-value**	**aOR (95% CI)**	**P-value**
Age				
>30 years	1.40 (0.48 – 4.11)	0.53	8.49 (1.09 – 65.75)	0.04
≤30 years				
Family atopy				
Yes	2.03 (0.71 – 5.78)	0.18	-	-
No				
Daily exposure to flour				
>8 hours	-	-	0.47 (0.17 – 1.30)	0.15
≤8 hours				
Work seniority				
6 – 10 years	6.53 (1.37 - 31)	0.01	1.13 (0.18 – 6.92)	0.8
>10 years	5.62 (1.22 – 25.9)	0.02	0.94 (0.16 – 5.4)	0.9
≤5 years				
Storage mite sensitization				
Yes	3.43 (1.38 – 8.51)	0.008	2.66 (0.87 – 8.18)	0.08
No				
Atopy				
Yes	2 (0.9 – 4.2)	0.07	2.46 (0.88 – 6.82)	0.08
No				
Sensitization to α-amylase				
Yes	1.54 (0.48 – 4.84)	0.46	-	-
No				
Wheat flour sensitization				
Yes	-	-	1.63 (0.52 – 5.08)	0.39
No				

aOR: adjusted odds ratio.

## Discussion

This study is among the first studies on bakery aeroallergen sensitization in sub-Saharan Africa. The prevalence of sensitization to flour and α-amylase found in this study were 16.6% and 8.3% respectively. After multivariate analysis, age, work seniority, and sensitization to storage mites were found as determinants of sensitization to occupational aeroallergens in the baking industry.

Wheat flour and α-amylase are known as major workplace aeroallergens in bakery [5]. In terms of prevalence of wheat flour sensitization, our finding is similar to 16% found by Baatjies et al. in South Africa [12]. The 3% prevalence of sensitization to α-amylase in their study was lower than that of our study. In western countries, studies show that 4% to 47% of bakery employees are sensitized to wheat flour and 4.6% to 20% of them are sensitized to α-amylase [10,18,19]. This large variability in proportion of subjects sensitized to inhalant allergens in bakeries across studies could be explained by the differences in the methods used in these studies. Some authors performed skin prick tests for the diagnosis of sensitization while others used the detection of serum-specific IgE antibodies. The difference in allergen exposure levels could also explain this difference of prevalence.

Other inhalant allergens in bakeries such as storage mites, mold and cockroaches although present in bakery are not considered as occupational allergens [5,14]. Storage mites have been unsuccessfully proposed as a workplace allergen because their prevalence of sensitization in bakers is similar to that observed in the general population [5,20]. In this study, 12.2% of employees were sensitized to storage mites. This finding corroborates those of other authors [14,21].

Many studies have investigated the factors associated with sensitization to wheat flour and α-amylase [10,13,14]. The most important determinant is atopy, with an odds ratio ranging from 3.7 to 20.8 [10]. After univariate analysis in our study, atopy and sensitization to storage mites were associated with both sensitization to wheat flour and α-amylase. In multivariate analysis, there was no longer a significant association between atopy and wheat flour, nor between atopy and α-amylase. Meanwhile positive skin prick tests to storage mites remained associated with sensitization to flour. De Zotti et al. also noted an association between positive skin prick tests to storage mites and sensitization to flour and to α-amylase [14]. Some authors believe that storage mites likely cause immunological co-sensitization and their positive skin prick tests are more an indicator of atopy rather than a response to an occupational allergen [13,22]. As found by several authors [13,15], in this study, work seniority was associated with sensitization to wheat flour [14,19]. Daily duration of exposure which is known to be an important factor of sensitization and asthma due to respiratory sensitizers [10,23,24] was not associated with sensitization to flour or α-amylase. This could be due to the fact that in our study, we did not measure exposure level. Cigarette smoking has a role in the underlying mechanism involved in occupational asthma. Through an IgE mediated response, its increases the risk of sensitization to high molecular weight aeroallergens that cause occupational asthma [25]. Gender and age have not been described as determinants of sensitization to these bakery aeroallergens [10]. In our study, age greater than 30 years was a determinant of sensitization to α-amylase. This can be explained by the fact that the work experience is more important in older people who have been exposed for a longer duration to the allergens.

Although this study contributes to the knowledge of respiratory allergy in bakers in a sub-saharan Africa setting, it has some limitations. The exposure to flour was not measured. In addition, the measurement of serum-specific IgE antibodies, a better method for the diagnosis of allergen sensitization could not be performed in participants. This weakness could have underestimated the prevalence of sensitization to wheat flour and α-amylase.

In conclusion, the prevalence of sensitization to major occupational aerollergens in Cameroon bakeries is significant. Work seniority, sensitization to storage mites and an age above 30 years, were the factors identified as associated with sensitization to work place inhalant allergens in bakery. Atopy was a potential risk factor. Skin prick tests to storage mites at the beginning of work in bakery could identify people at risk of developing sensitization to bakery aeroallergens. Future research could examine the preventive role of personal protective equipment and environmental hygiene with respect to sensitization to airborne allergens in baking industry.
